# Rice Husk Ash-Based Geopolymer Binder: Compressive Strength, Optimize Composition, FTIR Spectroscopy, Microstructural, and Potential as Fire-Retardant Material †

**DOI:** 10.3390/polym13244373

**Published:** 2021-12-14

**Authors:** Mohd Salahuddin Mohd Basri, Faizal Mustapha, Norkhairunnisa Mazlan, Mohd Ridzwan Ishak

**Affiliations:** 1 Department of Process and Food Engineering, Faculty of Engineering, Universiti Putra Malaysia (UPM), Serdang 43400, Malaysia; 2 Laboratory of Halal Science Research, Halal Products Research Institute, Universiti Putra Malaysia (UPM), Serdang 43400, Malaysia; 3 Laboratory of Biopolymer and Derivatives, Institute of Tropical Forestry and Forest Products (INTROP), Universiti Putra Malaysia (UPM), Serdang 43400, Malaysia; 4 Department of Aerospace Engineering, Faculty of Engineering, University Putra Malaysia (UPM), Serdang 43400, Malaysia; faizalms@upm.edu.my (F.M.); norkhairunnisa@upm.edu.my (N.M.); mohdridzwan@upm.edu.my (M.R.I.); 5 Institute of Advanced Technology (ITMA), Universiti Putra Malaysia (UPM), Serdang 43400, Malaysia

**Keywords:** rice husk ash, geopolymer, binder, mechanical properties, fire-retardant, compressive strength, stress-strain curve, ultimate stress, optimization, response surface methodology (RSM)

## Abstract

Compressive strength is an important property in construction material, particularly for thermal insulation purposes. Although the insulation materials possess high fire-retardant characteristics, their mechanical properties are relatively poor. Moreover, research on the correlation between fire-retardant and compressive strength of rice husk ash (RHA)-based geopolymer binder (GB) is rather limited. In addition, previous studies on RHA-based GB used the less efficient one-factor-at-a-time (OFAT) approach. In understanding the optimum value and significant effect of factors on the compressive strength, it was deemed necessary to employ statistical analysis and a regression coefficient model (mathematical model). The objective of the study is to determine the effect of different material behavior, namely brittle and ductile, on the compressive strength properties and the optimum material formulation that can satisfy both compressive strength and fire-retardant properties. The factors chosen for this study were the rice husk ash/activated alkaline solution (RHA/AA) ratio and the sodium hydroxide (NaOH) concentration. Compressive strength and fire-retardant tests were conducted as part of the experiments, which were designed and analyzed using the response surface methodology (RSM). The microstructure of geopolymer samples was investigated using a scanning electron microscope (SEM). Results showed that RHA/AA ratio was highly significant (*p* < 0.000) followed by NaOH concentration (*p* < 0.024). When the RHA/AA ratio was at 0.7 to 0.8 and the NaOH concentration was between 12 and 14 M, high compressive strength above 28 MPa was recorded. Optimum compressive strength of approximately 47 MPa was achieved when the RHA/AA ratio and NaOH concentration were 0.85 and 14 M, respectively. Brittle samples with low Si/Al ratio of 88.95 were high in compressive strength, which is 33.55 MPa, and showed a high degree of geopolymerization. Inversely, ductile samples showed low compressive strength and degree of geopolymerization. Water content within the geopolymer binder had a major effect on its fire-retardant properties. Semi-ductile GB showed the best fire-retardant properties, followed by semi-brittle and brittle GB. Using RHA as an aluminosilicate source has proven to be a promising alternative.

## 1. Introduction

Geopolymer is known for good mechanical properties and high early-aged strength. One of the properties is compressive strength. The potential advantages of rice husk ash (RHA)-based geopolymer material in enhancing its mechanical and thermal properties are mainly associated with rice husk (RH) with high silica content. Compared to fly ash (FA) or other aluminosilicate sources, RHA contained the highest silica between 85.0% and 95.0% and relatively low alumina content between 0.5% and 2.0%. Higher loading of RHA will result in higher silica content. Higher silica and lower alumina content mean higher silica to alumina (Si/Al) ratio. Therefore, higher mechanical strength can be achieved, as proved by Komnitsas and Zaharaki [1] and De Silva, et al. [2]. Although higher Si/Al ratio proved to provide higher compressive strength, a further increase in Si/Al ratio leads to a decrease in compressive strength, as studied by Songpiriyakij, et al. [3]. Geopolymer with a Si/Al ratio exceeding 24 may become more elastic and deform than brittle, failing under certain forces [4].

Songpiriyakij, et al. [3] studied the effect of the Si/Al ratio on compressive strength of FA-rice husk bark ash (RHBA)-based geopolymer binder. The increase in RHBA content will increase Si/Al ratio. After seven days of curing, higher Si/Al ratio resulted in higher compressive strength. At a Si/Al ratio of 15.91, the highest compressive strength of 54.90 MPa was recorded. The increase in RHBA content in the geopolymer binder enriched the matrix’s silica content, which created stronger Si–O–Si bonds. However, as the Si/Al ratio was further increased, the compressive strength dropped. It was probably due to early expansion and cracking of the binder specimen. Similar results were obtained by Fletcher, et al. [4], who studied the relationship between kaolinite-amorphous silica-based geopolymer binder and its compressive strength. Compressive strength increased together with Si/Al ratio, and the highest strength was recorded at 10.90 MPa with Si/Al ratio of 16. However, compressive strength started to drop when the Si/Al ratio was higher than 16. The failure mode was observed to change from crushing to deformation indicating a transformation in material behavior from brittle to ductile. 

De Silva, et al. [2] also studied the compressive strength of metakaolin-based geopolymer binder for Si/Al ratio ranging from 2.50 to 3.81. After 72 h of curing, higher silica content at Si/Al ratio of 3.81 resulted in higher compressive strength of 23.0 MPa. In a system with high Si concentration, condensation starts with the formation of oligomeric silicates, leading to poly (sialate-siloxo) and poly (sialate-disiloxo) forming three-dimensional (3D) rigid polymeric structures. Low Si content resulted in poly (sialate) polymer structures with high Si content and the formation of three-dimensional (3D) rigid polymeric networks. These 3D networks were formed from two structural units, which were poly (sialate-siloxo) and poly (sialate-disiloxo), which were built from oligomeric silicates. Due to the stable and more rigid 3D networks of poly (sialate-siloxo) and poly (sialate-disiloxo), samples with high Si content possessed higher compressive strength than those with low Si content. Based on the results, alumina content greatly influenced setting time, while silica content contributed to higher compressive strength.

Luna Galiano, et al. [5] conducted research on geopolymer binder composed of FA, metakaolin, and sodium silicate (Na_2_SiO_3_) in comparison with Ordinary Portland Cement (OPC). After 28 days of curing, OPC showed the highest compressive strength of 32.50 MPa, followed by FA-based geopolymer binder (G-FA) with 25.40 MPa and FA-metakaolin-based geopolymer binder (G-FA-MK) with 14.30 MPa. G-FA-MK exhibited the lowest compressive strength due to the lower amorphous phase of metakaolin (50.2%) compared to FA (72.3%). 

Madejska, et al. [6] studied the properties of geopolymer binder obtained from FA. Their objective was to study the influence of different concentrations, which are 10 M and 16 M of NaOH and KOH, on the compressive strength of the FA-based geopolymer binder. Their finding showed that higher concentration of OH at 16 M resulted in low compressive strength of geopolymer binder. Using NaOH of 10 M resulted in higher compressive strength (26.50 MPa) than KOH of 10 M (22 MPa). Other research on fly ash-based geopolymer was conducted by Kozub, et al. [7]. They investigated the compressive, flexural, and thermal radiation changes of geopolymer composites based on fly ash with the addition of melamine fibers in amounts of 0.5%, 1%, and 2% by weight. The results of the fire-jet test showed that the addition of melamine fibers resulted in a high temperature on the surface of the geopolymer plate. The visual observations made following the test reveal that the sample containing melamine has been significantly deteriorated. 

Kamarudin, et al. [8] also studied the relationship between the NaOH concentration (6, 8, 10, 12, and 14 M) and compressive strength of kaolin-based geopolymer binder after one, two, and three days of curing. The results showed that longer curing time resulted in higher compressive strength. Geopolymer binder with 12 M of NaOH showed the highest compressive strength of 5.90 MPa. Geopolymer binder with 14 M of NaOH showed slightly lower compressive strength, which is 5.50 MPa, than 12 M of NaOH due to an excess of sodium content in the binder. Geopolymer binder with 6 M of NaOH also recorded high compressive strength of 4.80 MPa after three days of curing due to higher water content, lower concentration, and ease of ions transportation during the geopolymerization process.

Buchwald, et al. [9] studied the properties of metakaolin-based geopolymer binder with and without fillers such as illitic clay, quartz fume, and recycled chamotte material. Their study concluded that the inclusion of illitic clay resulted in decreased compressive strength compared to geopolymer binder without filler which was 8.90 MPa. However, the addition of quartz fume and recycled chamotte material increased compressive strength to 11.00 MPa and 10.80 MPa, respectively. The results show that higher porosity of the binders will result in lower compressive strength and vice versa. 

Le, et al. [10] also studied metakaolin-based geopolymer. They investigated the temperature-dependent properties and fire resistance of metakaolin-based geopolymer foams. The results showed that the apparent density and drying shrinkage of the BGFs increased as the treatment temperature increased from 400 to 1200 °C. Below 600 °C, mass loss is increased while water absorption is decreased, and both parameters vary marginally between 600 and 1000 °C. The mass loss decreases significantly as the temperature rises above 1000 °C, although the water absorption increases. The compressive and flexural strengths of BGFs with high fiber are increased considerably at temperatures greater than 600 °C, with the highest values obtained at 1200 °C.

Cheng and Chiu [11] studied the relationship between the amount of silica in the geopolymer binder system and its compressive strength. Potassium hydroxide (KOH) and Na_2_SiO_3_ were used as activated alkaline (AA) solutions, and metakaolin and granulated blast furnace slag (BFS) were used as aluminosilicate sources. After one day of curing, the maximum compressive strength achieved was 70 MPa at a Si/Al ratio of 3.36. As the Si/Al ratio increased due to the addition of Na_2_SiO_3_, the strength gradually decreased. It was attributed to excess silicate, which hindered water evaporation and structure formation in the geopolymer system during the geopolymerization process.

Although some of the panel structures used as insulation materials should possess high fire-retardant characteristics, their mechanical requisites, including compressive strength for walls and doors, must not be unduly compromised. In addition, intensive research studies on the relationship between compressive strength and fire-retardant properties using a statistical analysis approach are not well established.

In addition, RHs are one of the largest available biomass resources, but their usefulness is rather limited. Despite RH weighing 20 to 25% of the harvested paddy and is a good source of renewable energy [12], only a marginal fraction is used [13], while the remainder is wasted through open burning or disposed of in landfills [14]. Improper handling of this voluminous organic waste may potentially pollute the environment, including the atmosphere (addition of carbon dioxide into the atmosphere through open burning, thus contributing to global warming), soil, and water

Despite many publications on RHA-based geopolymer composite reported in the literature in recent years [15,16,17,18,19], most studies were carried out in the one-factor-a-time (OFAT) approach. The novelty of this study is the application of statistical analysis and regression coefficients (mathematical models) to predict inquiries such as the optimal composition of geopolymer binder with improved fire-retardant and thermal properties to increase efficiency. Design of experiment (DOE) offers numerous advantages over the OFAT approach, including low resource requirements (experimental runs, time, materials, and human resources), accurate measurement of main effects and interactions, and the ability to analyze multiple variables at the same time. [20]. Furthermore, the RSM, initially coined by Box and Wilson [21], is widely used as a mathematical model for investigating significant effects, interactions, and optimization studies. Central composite design (CCD) is the most effective model for analysis and design [22]. Table 1 shows the difference in the total number of experimental runs between the full design of RSM and the full factorial (classical method) design based on 5-level factors. The full design of RSM requires only 31 experimental runs (one replication) for analyzing four factors, whereas a full factorial design requires 625 experimental runs. 

Since the RSM approach, specifically the CDD, has been widely used in geopolymer optimization [23,24,25,26], it was adopted in this study. The main objectives of this paper are to determine the effect of different material behavior, namely brittle and ductile, on the compressive strength properties and the optimum material formulation that can satisfy both compressive strength and fire-retardant properties.

## 2. Materials and Methods

### 2.1. Factors and Levels of the Design of Experiment (DOE) 

In the study, the ratio of RHA/activated alkaline solution (RHA/AA) and the sodium hydroxide (NaOH) concentration, designated as V_1_ and V_2,_ respectively, were chosen as factors. Other factors such as the ratio of AA solution, curing temperature, and curing time were kept constant at 5.5, 50 °C, and seven days, respectively. Factors and levels used in the DOE are shown in Table 2.

### 2.2. Design of Experiment

Five levels and two factors were applied in the central composite design (CCD) and three replications for 30 experimental runs at each design stage. The factors were selected based on preliminary lab work, their significant effect on the responses, and working range (workability). Table 3 displays the complete CCD with coded and uncoded levels of these factors. The value for the total block is 1, with the experiments carried out in randomized order. 

The optimization of RHA-based geopolymer binder was performed using Minitab @ 16.2 (Minitab, LLC, State College, PA, USA). An analysis of variance (ANOVA) was used to calculate the significance of the main factors and their interactions. The value of 95% was set as the significance level, which reflected the *p*-value of 0.05. Based on the correlation coefficient (R^2^) value, the regression coefficient model (mathematical model) developed in the ANOVA table was used for optimization purposes. To acquire the regression coefficient model, experimental data were fitted with the second-order polynomial model. The general mathematical model obtained from the analysis is shown in Equation (1),

(1) $$Y=\beta_{0}+\sum_{t=1}^{2}\beta_{i}X_{i}+\sum_{i}^{2}\beta_{ii}X_{i}^{2}+\sum_{i-1}^{1}\sum_{j=i+1}^{2}\beta_{ij}X_{i}X_{j}$$

where *Y* is the response, *β*_0_, *β_i_*, *β_ii_*, and *β_ij_* are regression coefficients for the intercept, linear, quadratic, and interaction terms, respectively. *X_i_* and *X_j_* are coded values for the independent variables [27].

### 2.3. Raw Materials and Sample Preparation

RHA was obtained from Maero Tech Sdn. Bhd (Nilai, Malaysia). It was ground using a planetary mill (Pulverisette 4, FRITSCH GmbH—Milling and Sizing, Idar-Oberstein, Germany) and sieved through 75 micron opening to obtain finer particle sizes. The fine structure of RHA before and after grinding was viewed under an S-3400N scanning electron microscope (SEM, Hitachi, Tokyo, Japan). The RHA particle size before grinding ranges from 1 μm to 100 μm. Particles appear as plates and thin shell-like structures with rectangular indents on the surface. These forms constitute the initial structure of the RH. RHA has porous, cellular surfaces due to its sponge-like particles. The particles contain a higher silica concentration in a natural solid-state, with amorphous shapes similar to cristobalite and trace crystalline quartz [28].

To develop pozzolanic activity, RHA was ground to a very fine particle size [29]. The condition for burning RH is vital in producing the highest silica RHA in an amorphous state. Conversely, silica derived from unchecked incineration (temperatures higher than 700 to 800 °C) comprises mainly cristobalite and tridymite, which are non-reactive silica minerals [30]. The physical properties of the RHA after grinding are given in Table 4.

NaOH and Na_2_SiO_3_ were purchased from Evergreen Engineering & Resources (Semenyih, Malaysia). The sodium-based solution was chosen over potassium-based, due to lower material cost [31] and better mechanical properties as reported in previous studies [32]. A sodium silicate solution was purchased from LGC Scientific Sdn Bhd (Selangor, Malaysia). The chemical composition (by wt.%) of the solution was Na_2_O = 11.9%, SiO_2_ = 57.8% and H_2_O = 30.3%. NaOH pellets with 97% purity were provided by Merck KGaA (Darmstadt, Germany). Different concentrations expressed as molarity, M of NaOH solution were prepared based on the number of pellets dissolved in de-ionized water.

**Figure 1:**
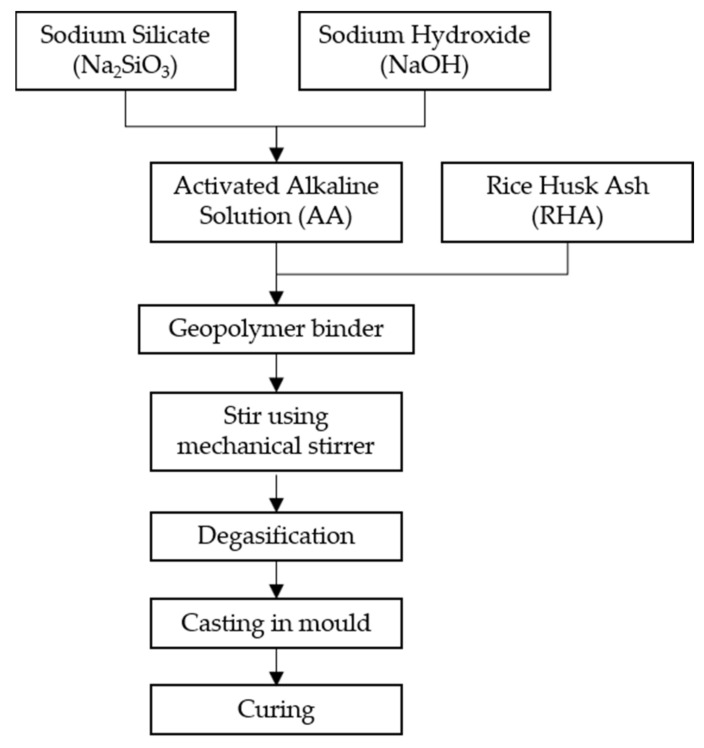
Flow chart for the fabrication of RHA-based geopolymer binder.

Samples were prepared according to the flowchart, as shown in Figure 1. Na_2_SiO_3_ was added into NaOH solution at a ratio of 5.5 to form an activated alkali solution (AA) solution. The solution was then mixed with RHA at a designated ratio (Table 3) to obtain a dark gray slurry mixture. The mixture was then stirred with a mechanical stirrer (HS-300, WiseStir, Thessaloniki, Greece) at 150 rpm for 30 min until homogenous. The mixture was then placed in a vacuum oven (Model 53, Binder GmbH, Tuttlingen, Germany) for degasification to remove the remaining tiny bubbles. The geopolymer binder was cast in mold and was placed in an oven to cure for 24 h at 50 °C. After 24 h, the samples were left at room temperature for six days or more (depending on the experimental design) for complete curing.

### 2.4. Compressive Strength Test

**Figure 2:**
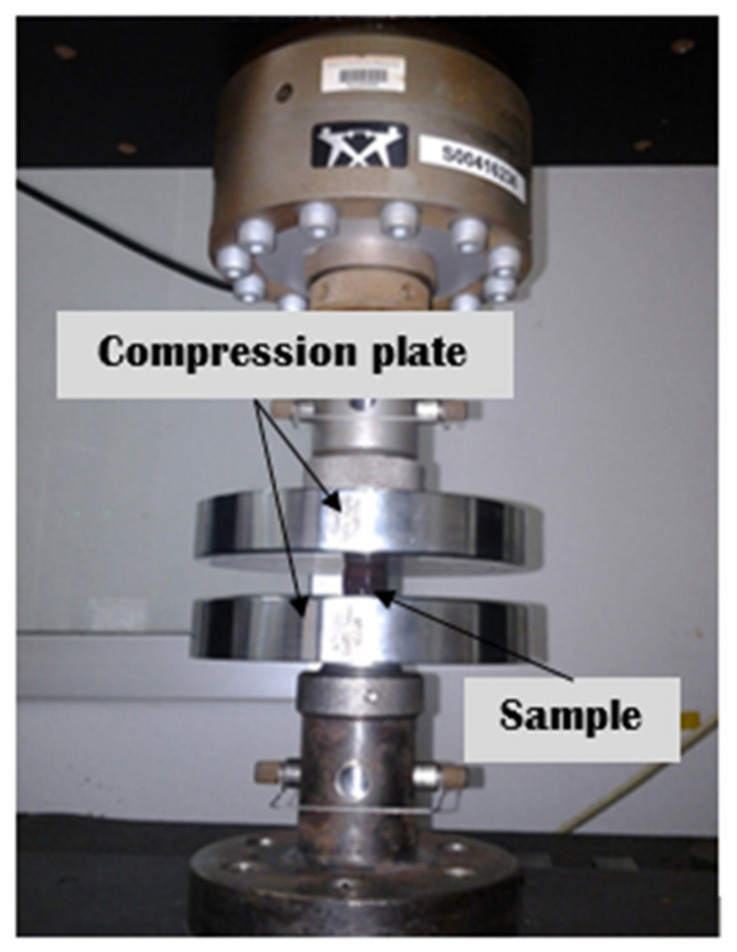
Set-up for compressive strength test.

The compressive test was conducted using the Instron 3382 Floor Model Universal Testing System according to the ASTM D695 standard [33]. Figure 2 shows the set-up for the compressive test where the sample was placed between the compressive plates and load was applied at a constant speed of 1.30 ± 0.13 mm/min. The test system has a load measurement accuracy of ±0.5% of reading.

**Figure 3:**
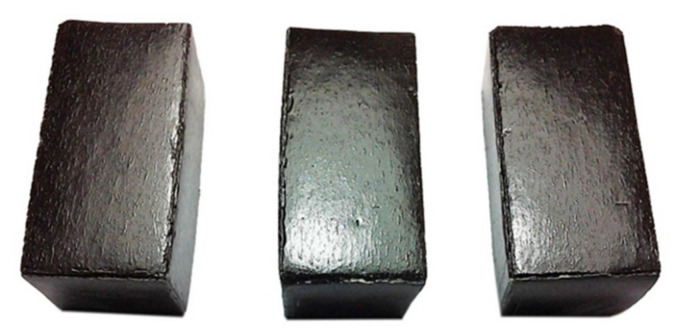
Specimens for the compressive strength test.

The present study was not solely focused on producing the highest compressive strength for geopolymer binder. However, adequate compressive strength was identified to determine the capability of the binder. The correlations between mechanical, thermal, and physical properties were studied as well. A rectangular test sample of dimensions (12.7 × 12.7 × 25.4) mm was preferred for this study based on the study conducted by Gyurkó and Nemes [34]. They found that the error in compressive strength decreases with the increase of the size of the sample. Additionally, cube samples are preferred as they have a higher deviation in strength ratio than the cylinder sample. Custom-made molds were used to obtain samples with flat and plane surfaces, as shown in Figure 3.

### 2.5. Fire-Retardant Test

The test was conducted by heating the samples with direct blow torch flame with flame temperatures around 900 °C. The samples were placed between the infrared camera and blow torch. The samples were kept 60 cm apart from the infrared camera and 7 cm from the blow torch. At the beginning of every test, the ambient temperature and humidity were recorded on the computer. Flame temperature versus time was taken as a result of fire protection in the experiment. The samples were exposed to direct flame for at least 20 min or until equilibrium temperature was reached.

### 2.6. Microstructure of Rice Husk Ash

**Figure 4:**
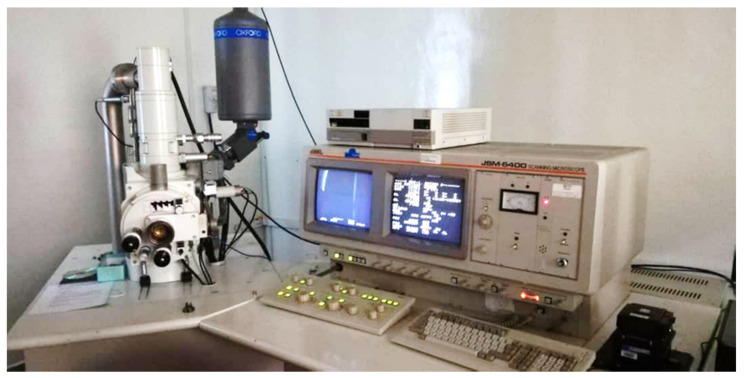
A scanning electron microscope (SEM).

A scanning electron microscope (SEM) (Hitachi, Tokyo, Japan) was used to analyze the difference in microstructure of samples after testing as shown in Figure 4. SEM was conducted using Hitachi S-3400N variable SEM. A total of four samples were taken, which is the sample exhibited ductile, semi-ductile, brittle, and semi-brittle. The samples were first mounted with a conductive adhesive and sputter-coated with gold-palladium powder. The stub with sample samples was inserted into the sample chamber of the SEM for viewing. Micrographs of sample surface were taken at magnifications of 250× and 5000×.

### 2.7. Fourier Transform Infrared Spectroscopy (FTIR)

FTIR allows infrared radiation to pass through a sample, forming a spectrum representing absorption peaks. FTIR spectroscopic measurements were conducted using Bruker VERTEX 70/70v FT-IR spectrometers (Bruker, Entlingen, Germany). In an air-purged sample chamber, all spectra were recorded at 4 cm^−1^, and 100 signal-averaged scans were obtained for each spectrum. The spectra and data were analyzed using OPUS software, and the samples were scanned from 4000 to 650 cm^−1^.

## 3. Results and Discussion

Size of RHA particles were in the range of 3.4 to 59.5 µm, with an average size aggregate of approximately 22.8 µm. The chemical composition of RHA is shown in Table 5. SiO_2_ was found to be the major constituent in RHA.

The complete design matrix and response values of compressive strength are given in Table 6. Data were analyzed using MINITAB.

### 3.1. Statistical Analysis of Compressive Strength

Table 7 shows the regression coefficients, the standardized coefficient error, and *p*-values of the effects of factors and their interaction. The results indicated that both factors and interaction effects were significant at a 95% confidence level. The *p* of regression analysis of RHA/AA ratio (V_1_) was highly significant (*p* < 0.000) followed by NaOH concentration (V_2_) with *p* < 0.024, while V_1_*V_2_ (interaction between both factors) produced the least significant effect (*p* < 0.034). Values for R^2^ = 0.9211 and R^2^ (adjusted) = 0.9085 were considered high, indicating that 92.11% of the sample variation in the response was attributed to the factors. Thus, the linear effect of factors was the major determining term that may cause significant effects on the response.

Equation (2) represent the regression models for the compressive strength, respectively.

(2) $$Y_{CS}=15.7+9.886\left(V_{1}\right)+1.421\left(V_{2}\right)+2.307\left(V_{1}V_{2}\right)$$

where *Y_CS_* represents the compressive strength, V_1_ and V_2_ are the decoded values of the RHA/AA ratio and NaOH concentration, respectively. The regression models can be used to calculate and analyze the effect of factors on the compressive strength performance of RHA-based geopolymer binder. 

### 3.2. Effect of Factors on Compressive Strength

**Figure 5:**
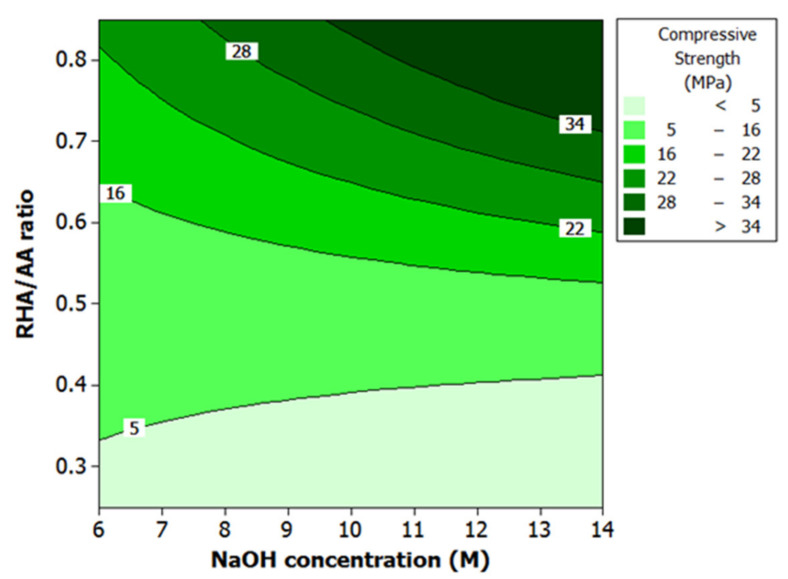
Contour plot for the effect of RHA/AA ratio and NaOH concentration on the compressive strength.

ANOVA and regression models were used to analyze the effect of various factors on the compressive strength of the material. Contour plots were used for better illustration. Figure 5 illustrates the effect of V_1_ and V_2_ on compressive strength. Compressive strength increased with higher values of both factors. At 0.7 to 0.8 for V_1_ and 12 M to 14 M for V_2_, higher compressive strength of more than 28 MPa was observed.

As the value of V_1_ and V_2_ increased, compressive strength also increased. The increase of V_2_ means that the mixture has less workability. Different aluminosilicate sources have different optimum strengths and workability. According to Kong, et al. [35], metakaolin-based geopolymer exhibited the optimum workability at an S/L ratio of 0.8, whereas an S/L ratio of 3.0 was optimal for FA-based geopolymer. RHA-based geopolymer had optimum workability similar to metakaolin at around 0.85. The low ratio indicated that RHA requires higher liquid demand than FA due to its finer particle size. 

The V_2_ also has a strong effect on compressive strength. However, the effect of V_2_ acted differently for the sample with brittle and ductile behavior. Figure 5 demonstrated that the sample with the RHA/AA ratio lower than 0.45 exhibited ductile behavior, while the sample with the RHA/AA ratio higher than 0.45 exhibited brittle behavior. For a sample with brittle behavior, a higher V_2_ produced higher compressive strength. At V_1_ of 0.8, a lower V_2_ of 6 M had compressive strength near 22 MPa. When V_2_ increased to 14 M, compressive strength was also elevated to over 34 MPa. 

Previous studies showed similar results when high V_2_ was used in geopolymer, which displayed brittle behavior. It was reported that high V_2_ of 12 M in FA-based geopolymer would promote rapid dissolution and hydrolysis, thus increasing compressive strength [36]. Other studies which used FA and bottom ash as aluminosilicate sources found that higher V_2_ of 10 M also produced higher compressive strength [37]. It was probably due to the higher dissolving rate of silica and alumina during geopolymerization. Rahim et al. (2014) also achieved the maximum compressive strength of 59.81 MPa when a high V_2_ of 12 M was used. 

**Figure 6:**
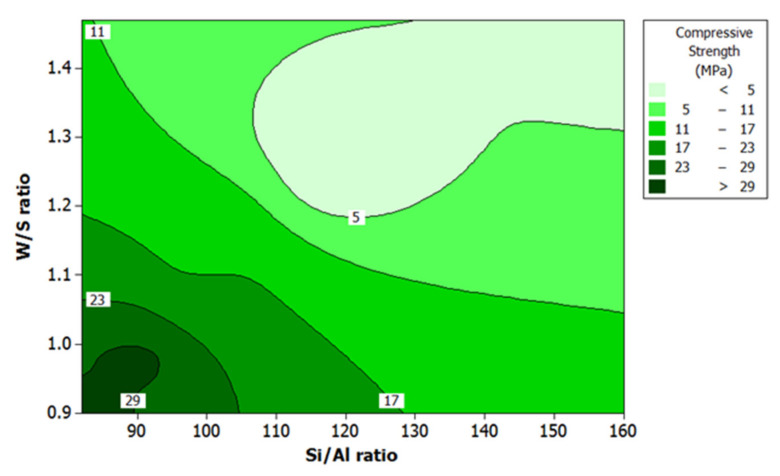
Contour plot of compressive strength, W/S ratio, and Si/Al ratio.

Higher RHA/AA ratio and lower Si/Al ratio will result in higher compressive strength, as shown in Figure 5 and Figure 6 respectively. High compressive strength of greater than 29 MPa was recorded when Si/Al ratio and W/S ratio were relatively low at 82 to 93 and 0.9 to 1.0, respectively. Since RHA/AA ratio is inversely proportional to the Si/Al ratio, this suggests that a higher RHA/AA ratio is associated with the lower Si/Al ratio. Figure 6 shows that lower W/S ratio within the range of 0.9 and 1.0 and Si/Al ratio within 82 and 93 resulted in higher compressive strength.

**Figure 7:**
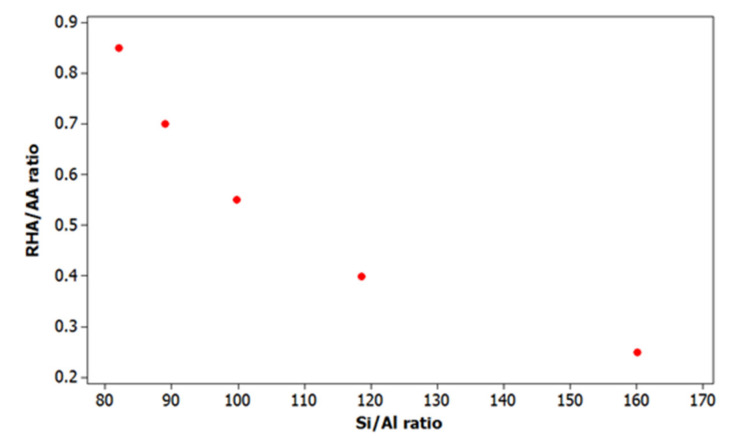
Relationship plot between RHA/AA ratio and Si/Al ratio.

Figure 7 shows the relationship between RHA/AA ratio and Si/Al ratio. The results were in agreement with the past research as reported by Temuujin, et al. [38]. The researchers found that a low Si/Al ratio of 1 gave a high compressive strength of 6.80 MPa compared to the higher Si/Al ratio of 2, which gave a lower compressive strength of 3.6 MPa. The depressed value was probably due to the high water content of the present mixtures [38,39].

### 3.3. Optimization of the Responses

**Figure 8:**
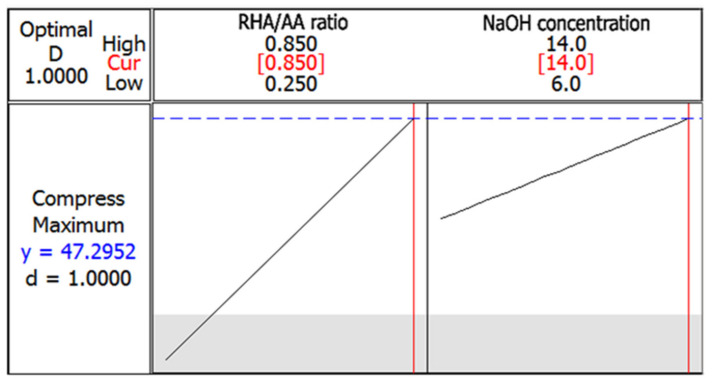
Optimization plot in compressive strength test.

Figure 8 shows the optimization plot and the effect of the different combinations of factor settings on the response. Both the lower and target values were set at 0 and 30 MPa, respectively. The maximum compressive strength of 47.30 MPa can be achieved when RHA/AA ratio (V1) is 0.85, and NaOH concentration (V_2_) is 14 M. The desirability of optimization was calculated as 1.0000, indicating that all parameters were within the target, which was to obtain the maximum compressive strength.

### 3.4. Experimental Validation

From Table 8, it was found that the average error for compressive strength was well below this value at only 1.82%. Three samples were tested to obtain the experimental value with a standard deviation of 0.90. It was concluded that the derived regression model established through this method could accurately optimize value for achieving compressive strength in the material. 

### 3.5. Material Behavior and Microstructural Analysis

Studies on the microstructural and material behavior were performed on four samples based on their compressive strength, as shown in Table 9. The samples were S23, S7, S28, and S5, which exhibited brittle, semi-brittle, ductile, and semi-ductile behavior, respectively. The selection was based on the stress-strain curves. Under the category of brittle material, samples S23 and S7 exhibited the highest and lowest compressive strength, respectively. Under the category of ductile material, samples S5 and S28 showed the highest and lowest compressive strength, respectively.

#### 3.5.1. Microstructural Analysis

**Figure 9:**
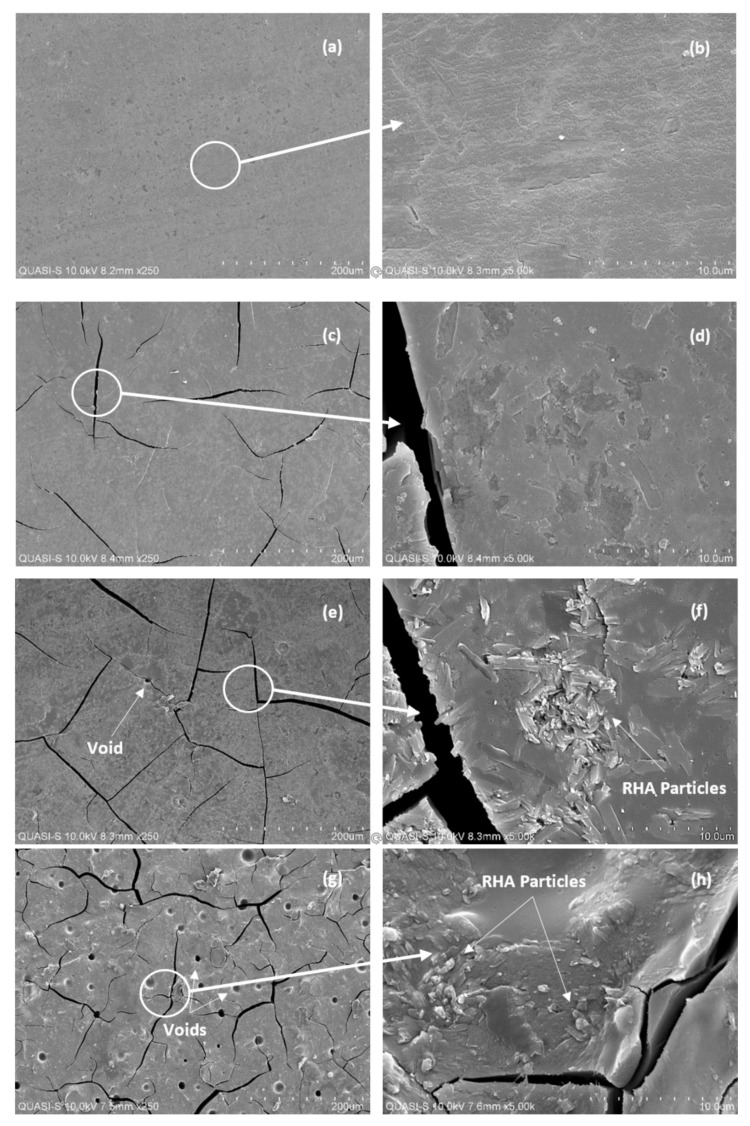
The SEM micrographs for sample S28—ductile (**a**,**b**), S5—semi-ductile (**c**,**d**), S7—semi-brittle (**e**,**f**), and S23—brittle (**g**,**h**).

Figure 9 shows SEM images of four geopolymer samples, namely S28, S5, S7, and S23, with different material behavior after the compressive test. Sample S28 showed that its amorphous matrix was homogenous with no obvious crystalline precipitates present as shown in Figure 9a,b. The material initiated no cracks and had a large plastic deformation range, enabling it to stretch a few times its original length. Sample S5, S7, and S23 initiated minor to major cracks due probably to the presence of voids in the structure of the geopolymer. 

During plastic deformation, voids will increase in number and grow bigger, while the sample density will decrease. Finally, the sample will fracture and form cracks due to the growth and coalescence of the voids [40]. From Figure 9c,d, sample S5 had a homogenous amorphous matrix with minimal unreacted RHA particles bonded in the matrix. Although no void can be seen on the surface of sample S5, microvoids were present, which initiated the cracks. Cracks formed in sample S5 were fewer and narrower compared to those in sample S7. 

Sample S7 and S23, as shown in Figure 9e–h, had a non-homogeneous amorphous matrix with some of the RHA particles remaining unreacted but were bonded in the matrix. However, the unreacted particles had only a minor effect on compressive strength since they did not act as fillers [41]. Compared to sample S7, the higher number and larger voids in S23 led to a wider and higher number of cracks formed.

The compressive strengths for each sample were 0.04 MPa (S28), 0.89 MPa (S5), 14.39 MPa (S7), and 34.60 MPa (S23). Samples S23 and S7 had the highest and lowest compressive strength in the brittle material category, respectively. Samples S5 and S28 had the highest and lowest compressive strength in the category of ductile material, respectively. Ductile materials are those that can undergo extensive plastic deformation under tensile stress prior to rupturing. In other words, it will stretch when subjected to tensile force. Ductile fracture is caused by plastic deformation of the material at the crack tip. This frequently results in a steady and predictable form of fracture in which crack propagation can occur only with increasing applied force. Conversely, brittle materials break without significant plastic deformation under tensile stress and called sudden failure. Brittle fracture is characterized by the development of cracks with little or no ductile deformation of the material surrounding the crack tip. This is an undesirable mode of fracture because, when a critical load is achieved, brittle cracking can cause the material to break completely in a relatively short time. 

**Figure 10:**
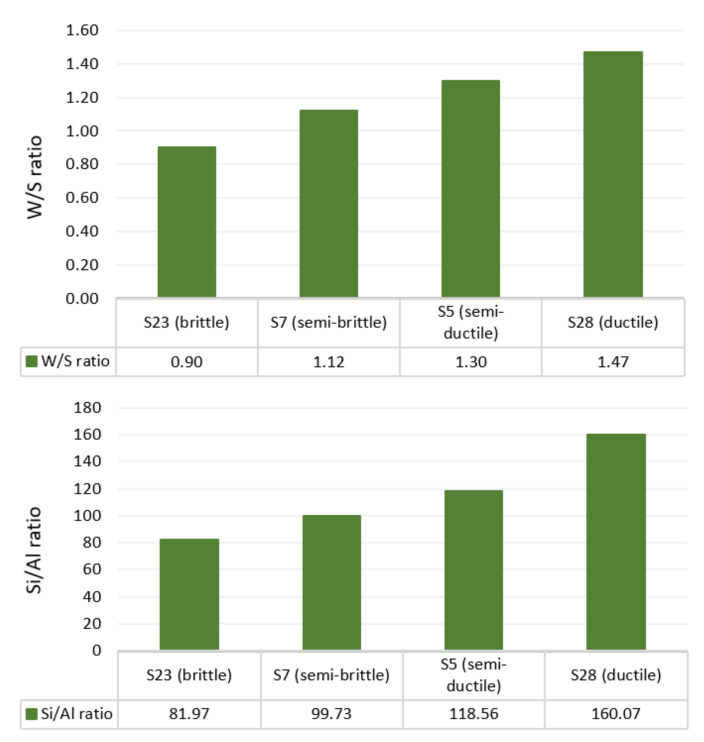
W/S ratio and Si/Al ratio in selected brittle and ductile samples.

Semi-brittle material such as S7 possessed lower compressive strength compared to the brittle sample S23. The behavior of the former material was due to its lower viscosity caused by higher water content and Si/Al ratio, as seen in Figure 10. A semi-ductile material, such as S5, possessed higher compressive strength due to lower water content and Si/Al ratio than fully ductile material S28.

#### 3.5.2. Material Behavior

**Figure 11:**
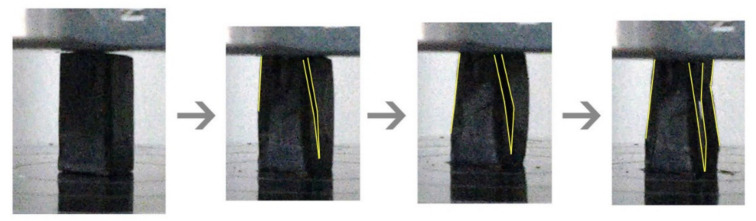
Cracking or fracturing process of brittle material (sample S23) under compression.

Based on the stress-strain curve obtained, samples can be classified as either brittle or ductile. Different ratios of Na_2_SiO_3_, NaOH, and RHA and temperature produce different material properties. Brittle materials such as concrete, stone, and ceramic showed slight contraction after the proportional limit was exceeded. Maximum compressive strength is at the highest point where the sample fails with further stress. When stress reached yield point, the strain started to increase faster than stress. It causes cracks as shown by lines to form, which will grow wider, thus causing the sample to fail, as shown in Figure 11. 

**Figure 12:**
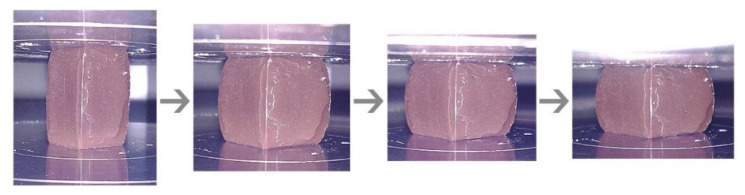
Ductile material (sample S5) during compressive strength.

Typical ductile materials, such as steel, aluminum, and copper, have a stress-strain curve identical to that of brittle materials until the yield point. It exhibits linear elasticity at an early stage when the cell walls begin to bend. With greater applied force, the material undergoes a transition from elastic to plastic behavior. In this plastic region, the material is deformed permanently and able to endure approximately constant stress with increasing compressive strain. The cell walls of the material start to buckle and usually becomes barrel-shaped. Further force applied resulted in the cell walls begin to collapse and compact together, and further compressive stress will increase exponentially with strain. Figure 12 shows ductile material undergone shape deformation (barreling) during the compressive test.

**Figure 13:**
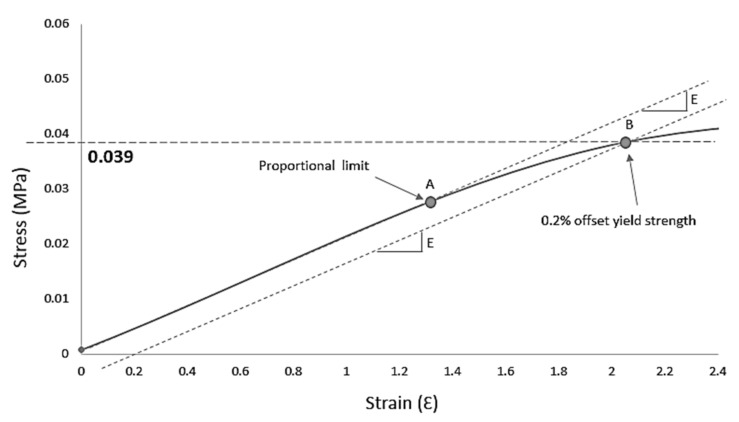
Example of construction of 0.20% offset yield strength for sample S28.

There is no single clear point of failure that can be identified until the yield point. Since it is difficult to determine the exact point where plastic deformation begins, the offset method is often used to calculate the yield strength at 0.01, 0.10, 0.20, or 0.50% offset of the sample with an equal slope compressive modulus. In this study, a typical 0.20% offset was chosen, and the offset yield strength was constructed, as shown in Figure 13. A line that is drawn from the zero points and tangent to the curve is called proportional limit or elastic limit, and its slope (E) is called young’s modulus. At point A, the material will recover its original shape when the applied force is removed. If the force is applied beyond this point, the material will experience plastic behavior when permanent deformation occurs. A parallel line is drawn from the strain axis at Ԑ_e_ = 0.20% and intersect with the curve line at point B. The value of stress at point B represents the compressive strength of the sample. 

**Figure 14:**
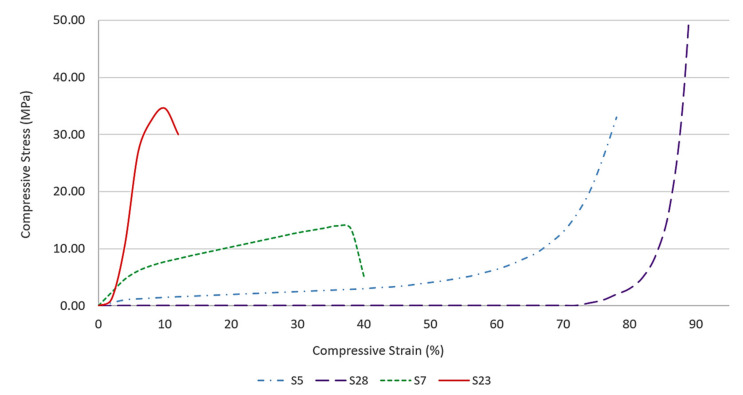
Stress-strain curves of sample S5 (semi-ductile), S28 (ductile), S7 (semi-brittle), and S23 (brittle).

Figure 14 shows the stress-strain curve for brittle and ductile samples. Sample S23, which exhibited brittle behavior, failed faster than that of sample S7. Although sample S23 was able to withstand high stress, the sample was brittle, and further stress resulted in cracks propagation. Sample S7, which exhibited semi-brittle behavior, recorded lower maximum compressive strength. However, the sample can deform without fail at four times more than sample S23. It is beneficial for application which both strength and flexibility. On the other hand, sample S7, which exhibited semi-brittle behavior, recorded higher compressive strength than sample S5. Sample S5 did not exhibit any crack up to 50% compressive strain. Further stress resulted in the sample to form cracks at the edge of the sample. Sample S5 and S28 were categorized under ductile material as they formed barrel shapes during the plastic region. Beyond the plastic region, only sample S28 did not form any crack. However, the sample was completely flat. 

In addition, a steeper slope indicates that the material is more resilient and not easily deformed than a gentler slope. It showed that sample S23 (V_1_ = 0.85, V_2_ = 10 M) had the steepest slope, indicating it is the most resilient in resisting deformation than samples S7 (V_1_ = 0.55, V_2_ = 10 M). Among ductile samples, S5 (V_1_ = 0.40, V_2_ = 8 M) and S28 (V_1_ = 0.25, V_2_ = 10 M) had the steepest and shallowest slope, respectively. In conclusion, ductile samples exhibited shallower slopes relative to brittle ones indicating that these samples were easily deformed due to lesser resilience [42].

### 3.6. FTIR Spectroscopy Characterization

**Figure 15:**
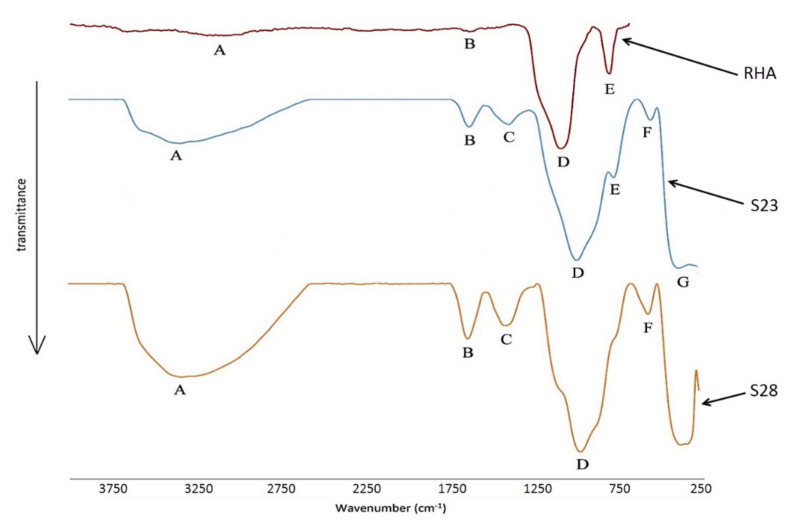
FTIR spectra of RHA, sample S23, and S28.

Compressive strengths of the hardened geopolymeric matrices, made with different mixture proportions, showed considerable differences. Two geopolymer samples were chosen with different material behavior, namely S28 (ductile) and S23 (brittle). The FTIR technique was employed to characterize the presence of a functional group of these hardened geopolymeric pastes. RHA was used as a control. The FTIR spectra of the RHA, samples S28 and S23, are shown in Figure 15. Table 10 illustrated the characteristic bands of each spectrum according to Chindaprasirt, et al. [37] and Fernández-Jiménez and Palomo [43]. 

Significant broad bands were observed at the wavenumber range of 3453 to 3153 cm^−1^ (band A) and 1660 to 1630cm^−1^ (band B) for O–H stretching and O–H bending, respectively. The wavenumber range of 1424 to 851 cm^−1^ (bands C and D) and 797 to 786 cm^−1^ (band E) were attributed to the asymmetric stretching vibration of Si–O/Al–O and Si–O–Si stretching quartz, respectively.

Additionally, the wavenumber range of 580 to 568 cm^−1^ (band F) was associated with the presence of zeolites, as discussed in previous studies [43,44,45]. According to Król and Jeleń [46], the stretching vibrations of double four-membered rings in the LTA structure were characterized by a band in that range that was symmetrically stretched. This band was a superposition of multiple component bands in the spectrum of the zeolite, as shown by its complex envelope. The calculated spectra revealed that the normal ring vibration (PO—pore opening vibration) produced a band at lower wavenumbers. The presence of this band in the investigated spectra suggested that this unit (double four-membered ring) was stable across the temperature range studied. 

The distinct wave number range of 399 to 383cm^−1^ (band G) was ascribed to the O–Si–O bending mode of SiO_4_ tetrahedra. According to Fernández-Jiménez and Palomo [43], the intensity of this band corresponds to the degree of amorphization of the material, whereas the intensity of the band does not correspond to the degree of crystallization of the material.

Using RHA as a reference, the band at approximately 1087cm^−1^ shifted to a range of 1003 to 851cm^−1^ after the samples reacted with the AA solution. This result has been discussed by Van Jaarsveld, et al. [47]. These values shifted to lower wavenumbers when the degree of silicon substitution by aluminum in the second coordination sphere increased due to the weaker Al–O bond, as previously observed by Wei and Zongjin [48]. 

Furthermore, the Si–O–Si position at band D shifted to a lower frequency than that of the original ashes. When the SiO_4_ tetrahedron is partially replaced by AlO_4_ tetrahedron during the hydration reaction, a change in the local chemical environment of the Si–O bond occurs. It may explain the large shift towards the low wavenumber. [48]. As observed in a previous study [43], these displacements indicated that new products were formed from the reaction between the ashes and the AA solution, as shown in band D as an example. Chen, et al. [49] determined that this decrease in intensity indicated that the amorphous phase in the ashes was depolymerized to Si–O and Al–O bonds, while the shifts suggested polycondensation of these bonds in the alkaline environment.

According to Chindaprasirt, et al. [37], Si–O–Si stretching vibration is more prominent than the O–Si–O bending mode. Therefore, it is logical to use Si–O–Si stretching vibration to assess the degree of geopolymerization. Peak area and peak height are frequently used in the quantitative assessment of geopolymer reaction. The ratio of inverted peak height (H) and the ratio of the area of the inverted peak (AS) of Si–O–Si stretching vibration are tabulated in Table 11. The peak height ratio (H ratio) is calculated by dividing the inverted height of the samples (S23 or S28) with RHA at band D of Si–O/Al–O stretching. Compared to the H value, the value of the AS ratio gives a better reflection of the degree of geopolymerization [37].

For the RHA system, the AS was set to 1.00, which is the lowest value indicating zero degrees of geopolymerization. Compared to the RHA sample, samples S23 and S28 had a higher AS and H ratio associated with an increase and broadening in the IR intensity of IR peaks, indicating a higher degree of geopolymerization. A higher rate of geopolymerization was associated with a lower Si/Al ratio. The result was in agreement with López, et al. [50], who similarly concluded that higher content of RH silica (higher RHA/AA ratio) would result in a higher degree of geopolymerization. 

In addition, the spectral data have a broader peak at the 3453 to 3330cm^−1^ range for OH stretching in Si∓OH and Al∓OH. It was apparent that the geopolymer derived from RHA tended to have a broad OH stretching peak. It can be inferred that higher RH silica content may facilitate geopolymerization with RHA. Both samples S23 and S28 had a relatively high AS and H ratio, indicating that both samples underwent relatively high geopolymerization. However, higher geopolymerization will not necessarily result in higher compressive strength, as reported in previous studies [37,51]. 

The type of sample material, either ductile or brittle, should be taken into account. Ductile material with a higher Si/Al ratio and W/S ratio will exhibit low compressive strength despite having a higher degree of geopolymerization due to high water content. However, with brittle samples, which are S23, AS and H ratio corresponded very well with compressive strength, as shown in Table 11. Higher AS and H values produced higher compressive strength

### 3.7. Potential as Fire-Retardant Material

As reported in the literature review, geopolymer, which produced good mechanical properties, such as high compressive strength, failed to exhibit good thermal properties such as fire-retardant. This study also arrived at a similar conclusion. However, the study also suggested that semi-brittle and semi-ductile materials may have wide options for industrial applications in mechanical and thermal properties. All samples tested exhibited intumescent behavior. According to Ullah, et al. [52], when exposed to heat, intumescent materials expand by forming a multicellular layer. This layer effectively protects the substrate from very high temperatures, thus helping maintain the substrate’s structural integrity.

**Figure 16:**
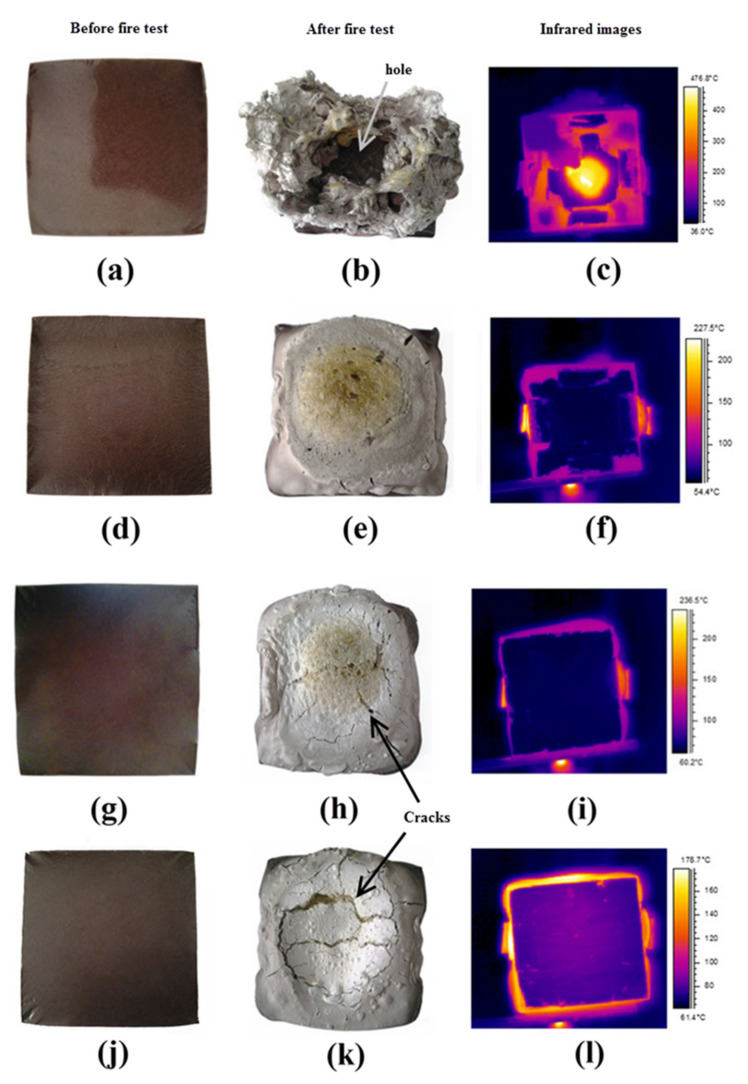
Images of RHA-based geopolymer binder and infrared images before and after fire-retardant test for samples S28 (**a**–**c**), S5 (**d**–**f**), S7 (**g**–**i**), and S23 (**j**–**l**).

Sample S28, which exhibited ductile behavior, has poor compressive strength and fire-retardant, as shown in Figure 16a–c. One of the major reasons for this is the high water content. As shown in Figure 10, the sample contained the highest water content giving a glossy surface to the geopolymer panel before being exposed to fire. When the sample was heated rapidly to a high temperature, part of its water content, in the form of bound or absorbed water, evaporated. The pressure developed simultaneously in the pores of the material vacated by the evaporation. 

According to Luna Galiano, et al. [5], evaporated water molecules supposedly travel from the hot fire-exposed surface to the inner part of the material, which was cooler. However, the material pores expanded faster due to the rapid evaporation rate, resulting in a weak matrix structure and the release of the evaporated water from the material. The intumescent process could not occur simultaneously as the geopolymer surface could not crystallize in time. With the failure of the geopolymer system, heat and fire were able to travel through the panel matrix and create a hole. The infrared images in Figure 16 show the temperature at the center of the sample immediately before the final layer of the geopolymer panel failed and was subsequently penetrated by the fire. 

**Figure 17:**
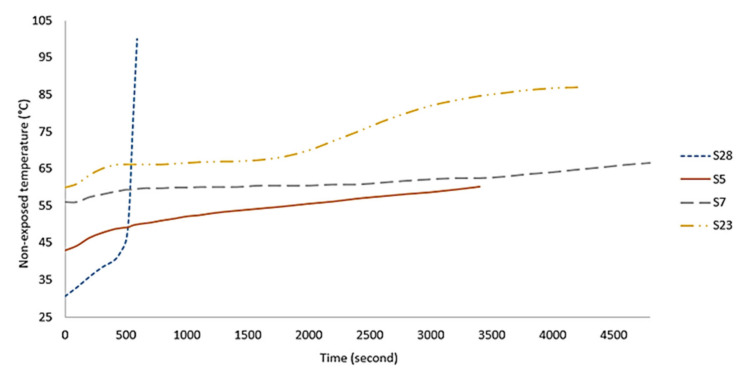
Time-temperature curves of selected geopolymer binder samples.

Sample S5, which shows semi-ductile behavior produced the best fire-retardant properties with a maximum non-exposed surface temperature of only 50 °C after 50 min, as shown in Figure 17. No cracks or holes were formed after the fire test, indicating that the sample exhibited a proper intumescent process. A portion of the water content evaporated when exposed to the fire and generated pressure in the pores of the material and microvoids. Since crystallization of the geopolymer surface and intumescent process successfully took place in the sample, the evaporated water molecules were transported to a cooler area of the material, resulting in very low temperature at the non-exposed area [5].

Sample S7 in Figure 16g–i, which showed semi-brittle behavior, also possessed good fire-retardant properties. This sample had a similar process as that in the semi-ductile S5. The only difference was small voids in S7, which initiated cracks when the voids and pores expanded under pressure created by evaporated water. The surface cracks directly in contact with the fire allowed heat to enter and penetrate to a cooler area in the material, thus raising the non-exposed surface temperature. The non-exposed surface temperature is the temperature at the opposite side of the sample. Sample S23, which exhibited brittle behavior, also displayed a similar process as S7 and S5. 

**Figure 18:**
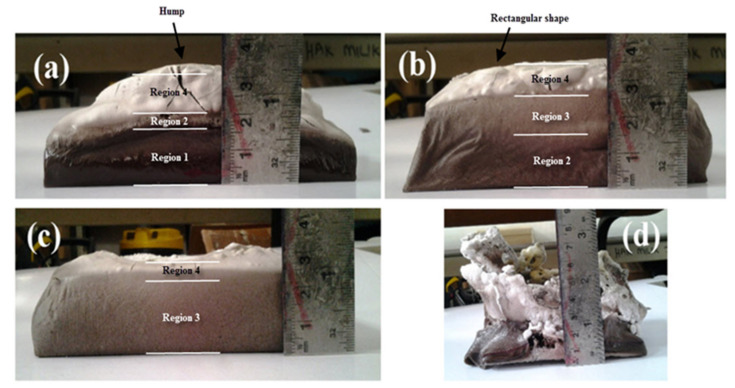
Color difference in binder layer of geopolymer sample (**a**) S5, (**b**) S7, (**c**) S23, and (**d**) S28.

However, due to more voids present in the geopolymer material and wider crack openings generated during the fire test, a higher amount of heat can penetrate and dissipate in the material resulting in a higher non-exposed surface temperature of around 85 °C after 50 min. With further propagation of surface cracks, the exposed geopolymer structure began to collapse, especially at the center of the material. Figure 18 shows the different phases displayed by the geopolymer panels during the fire test.

Sample S28 failed as a fire-retardant panel due to the very high content of water. The geopolymer binder lost water through evaporation more rapidly than its rate of crystallization. The remaining S5, S7, and S23 samples underwent the intumescent process and showed potential as good fire-retardant geopolymer panel material. After fire tests, all samples tested expanded at different rates and developed thicknesses between 30 and 35 mm, an expansion of approximately three times its original thickness. 

**Figure 19:**
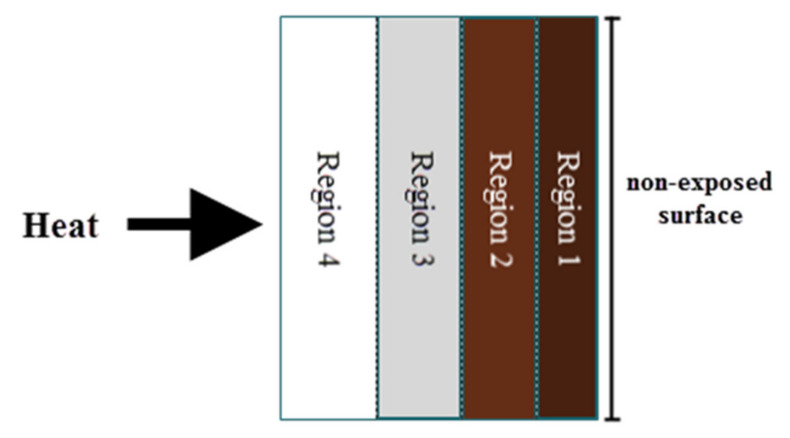
Diagram of color variation across geopolymer panel after fire-retardant test.

There was also a change in color across the thickness of the samples, which was most likely caused by different phase transformations occurring within the material due to exposure to a high flame temperature between 850 and 900 °C for an extended time. The color changes were not uniform and can be divided into four regions, region 1 (dark brown), region 2 (light brown), region 3 (gray), and region 4 (white), as shown in Figure 19. 

Region 1 was the region where geopolymer binder remained unchanged. The heat temperature at this region is too low to initiate mineralogical transformations due to the small amount of heat that passes through this region, as indicated by a slight increase in non-exposed temperature, as shown in Figure 17. Sample S5, which had a large portion of region 1 at the non-exposed surface, is the best geopolymer panel with optimum mixing composition due to its lowest heat transfer. 

Region 2 showed light coloration due to higher temperature in this region compared to region 1. This color change is attributed to the oxidation changes in the Fe_2_O_3_ of the RHA, as reported by Temuujin, et al. [38]. Despite the absence of region 1, sample S7 produced good fire-retardant properties since it had region 2 at the non-exposed surface. The higher non-exposed surface temperature of S23 may be due to the large portion of region 3. The temperature in region 2 and 3 is high enough to initiate mineralogical transformations and are thus reflected in the colored layers.

Fire-retardant capacity can also be gauged from the expansion of geopolymer. Some of the samples mainly expand centrally, producing a humped surface such as in sample S5. Others, such as samples S7 and S23, expanded equally in all directions forming a rectangular shape. The central hump in S5 showed that heat was limited to the center thus indicating low thermal diffusivity beyond that area. The material showed good fire-retardant to block heat from spreading to other parts, not in direct flame contact. 

## 4. Conclusions

Study on the compressive strength, optimize composition, FTIR spectroscopy, microstructural and potential as fire-retardant material of RHA-based geopolymer binder (GB) has been successfully performed. Response surface methodology (RSM) was successful in identifying the significant factors and optimizing the responses. Results for the compressive strength showed that RHA/AA ratio was highly significant (*p* < 0.000), followed by NaOH concentration (*p* < 0.024). When the RHA/AA ratio was at 0.7 to 0.8 and the NaOH concentration was between 12 and 14 M, high compressive strength above 28 MPa was recorded. When the RHA/AA ratio and NaOH concentration were 0.85 and 14 M, respectively, the compressive strength was approximately 47 MPa. Brittle samples with a low Si/Al ratio of 88.95 demonstrated a high compressive strength of 33.55 MPa and a high degree of geopolymerization. In contrast, ductile samples exhibited low compressive strength and a low degree of geopolymerization. The amount of water in the geopolymer binder had a significant impact on its fire-retardant properties. Semi-ductile GB performed the best in terms of fire retardancy, followed by semi-brittle and brittle GB. The use of RHA as an aluminosilicate source has proven to be a viable option. The RHA-based GB, which samples demonstrated good fire-retardant (burn slowly and thermally stable) and mechanical properties, has enormous potential for use as an insulation material in various fields. 

## Figures and Tables

**Table 1:** Total number of experimental runs for full factorial design and RSM based on 5-level factors.

Design	Factors					
	2	3	4	5	6	7
RSM (CCD)	13	20	31	52	90	152
Full factorial design	25	125	625	3125	15,625	78,125

**Table 2:** Factors and levels.

**Factor**	**Unit**	**Notation**	**Levels**				
			−2	−1	0	1	2
RHA/AA ratio	-	V_1_	0.25	0.40	0.55	0.70	0.85
NaOH concentration	M	V_2_	6	8	10	12	14

**Table 3:** Design matrix.

Sample	Coded Factor		Uncoded Factor	
	V_1_	V_2_	V_1_	V_2_
S1	−1	−1	0.40	8
S2	1	1	0.70	12
S3	1	1	0.70	12
S4	1	−1	0.70	8
S5	−1	−1	0.40	8
S6	−1	1	0.40	12
S7	0	0	0.55	10
S8	1	1	0.70	12
S9	0	0	0.55	10
S10	−1	1	0.40	12
S11	0	0	0.55	10
S12	1	−1	0.70	8
S13	−1	−1	0.40	8
S14	0	0	0.55	10
S15	0	0	0.55	10
S16	−1	1	0.40	12
S17	1	−1	0.70	8
S18	0	0	0.55	10
S19	0	−2	0.55	6
S20	2	0	0.85	10
S21	−2	0	0.25	10
S22	0	2	0.55	14
S23	2	0	0.85	10
S24	0	−2	0.55	6
S25	−2	0	0.25	10
S26	0	2	0.55	14
S27	2	0	0.85	10
S28	−2	0	0.25	10
S29	0	2	0.55	14
S30	0	−2	0.55	6

**Table 4:** Physical properties of RHA after grinding.

Properties	RHA
Particles Size	<75 µm
Color	Light gray
Structure	Powder
Odor	Non

**Table 5:** Chemical composition of RHA.

Component.	SiO_2_	PdO	Al_2_O_3_	Fe_2_O_3_	CaO	K_2_O	Cr_2_O_3_	MnO	NiO	CuO	ZnO
Mass (%)	87.40	6.00	3.00	1.49	1.40	0.49	0.27	0.19	0.07	0.05	0.04

**Table 6:** Design matrix and response value for the compressive strength.

Sample	RHA/AA Ratio (V_1_)	NaOH Concentration (V_2_)	RHA/AA Ratio (V_1_)	NaOH Concentration (V_2_)	Compressive Strength (MPa)
S1	−1	−1	0.40	8	0.80
S2	1	1	0.70	12	31.07
S3	1	1	0.70	12	33.55
S4	1	−1	0.70	8	23.20
S5	−1	−1	0.40	8	0.89
S6	−1	1	0.40	12	0.48
S7	0	0	0.55	10	14.39
S8	1	1	0.70	12	33.32
S9	0	0	0.55	10	13.82
S10	−1	1	0.40	12	0.65
S11	0	0	0.55	10	15.10
S12	1	−1	0.70	8	25.49
S13	−1	−1	0.40	8	1.03
S14	0	0	0.55	10	13.31
S15	0	0	0.55	10	14.88
S16	−1	1	0.40	12	0.64
S17	1	−1	0.70	8	22.52
S18	0	0	0.55	10	15.34
S19	0	−2	0.55	6	16.12
S20	2	0	0.85	10	30.97
S21	−2	0	0.25	10	0.05
S22	0	2	0.55	14	20.29
S23	2	0	0.85	10	34.60
S24	0	−2	0.55	6	15.53
S25	−2	0	0.25	10	0.04
S26	0	2	0.55	14	17.78
S27	2	0	0.85	10	30.19
S28	−2	0	0.25	10	0.04
S29	0	2	0.55	14	21.90
S30	0	−2	0.55	6	15.64

**Table 7:** Estimated effects and coefficient for RHA/AA ratio and NaOH concentration on the compressive strength.

Term	Notation	Coefficient	Std. Error of Coefficient	*p*-Value
Constant		15.700	0.6632	0.000
RHA/AA ratio	V_1_	9.886	0.5932	0.000
NaOH concentration	V_2_	1.421	0.5932	0.024
RHA/AA ratio*NaOH concentration	V_1_ * V_2_	2.307	1.0274	0.034
R^2^ = 0.9211 R^2^ (adj) = 0.9085				

**Table 8:** Experimental validation for the compressive strength.

Sample	Compressive Strength (MPa)		
	Experimental Value	Predicted Value	Error (%)
SV1	48.91	47.30	3.40
SV2	48.10	47.30	1.69
SV3	47.12	47.30	0.38
	$\bar{x}$ Error		1.82

**Table 9:** Experimental validation for the compressive strength.

Sample	RHA/AA Ratio (V_1_)	NaOH Concentration (V_2_)	Compressive Strength (MPa)
S5	0.40	8	0.89
S7	0.55	10	14.39
S23	0.85	10	34.60
S28	0.25	10	0.04

**Table 10:** Characteristic bands of each spectrum in RHA and sample S23, and S28.

Band	RHA (cm^−1^)	S23 (cm^−1^) Brittle	S28 (cm^−1^) Ductile	Characteristic Bands
A	3153	3343	3330	O–H stretching (H_2_O)
B	1630	1638	1643	O–H bending (H_2_O)
C	-	1406	1424	Si-O/Al-O stretching
D	1087	1003	978	Si–O/Al–O stretching
E	797	786	-	Si–O–Si stretching quartz
F	-	568	580	Zeolites
G	-	-	383	O–Si–O bending (SiO_4_)

**Table 11:** Inverted peak area and AS ratio from FTIR spectra of RHA and geopolymer samples at Si–O–Si stretching vibration in a compressive test.

Sample	Compressive Strength (MPa)	Location of Si–O–Si (cm^−1^)	AS Ratio	H Ratio	Si/Al Ratio	W/S Ratio
RHA	-	1087	1.00	1.00	-	-
S23	33.55	1003	2.01	1.25	88.95	0.26
S28	0.04	978	2.01	1.39	160.07	0.41

## Data Availability

The data presented in this study are available on request from the corresponding author.
